# Pilot study of blood perfusion coherence along the meridian in forearm

**DOI:** 10.1186/1472-6882-13-327

**Published:** 2013-11-23

**Authors:** Guangjun Wang, Yuying Tian, Shuyong Jia, Wenting Zhou, Weibo Zhang

**Affiliations:** 1 Institute of Acupuncture and Moxibustion, China Academy of Chinese Medical Sciences, Beijing, China

**Keywords:** Coherence analysis, Full-field laser blood perfusion imaging, Meridian, Age, Inflatable occlusion

## Abstract

**Background:**

Many studies have explored the relationship between skin microcirculation and meridian activation. However, few studies have examined blood perfusion coherence along the meridians, and other studies have suggested that the skin vasodilator response relates to age. This study investigated blood perfusion coherence characteristics along the meridian of the forearm in healthy volunteers.

**Methods:**

A total of 15 young subjects (25.53 ± 2.20) and 15 middle-aged subjects (50.07 ± 3.37) were recruited for this study. Before experiments, each subject was placed in a temperature-controlled room for 60 min. Skin blood perfusion from five points was recorded simultaneously using a full-field laser perfusion imager before and after inflatable occlusion. The five points comprised three points located on the pericardium meridian, and two points from different locations. Coherence analysis between these points was performed at different frequency intervals from 0.0095 to 2 Hz.

**Results:**

In young subjects, the coherence value was unchanged before and after occlusion, and there was no significant difference in coherence value between meridian-meridian points (M-M) and meridian-parameridian points (M-P). In middle-aged subjects, the coherence value increased significantly in both M-M and M-P at frequency intervals of 0.14-0.4 Hz, 0.4-1.6 Hz, and 1.6-2 Hz. However, there was no significant difference in coherence values between M-M and M-P.

**Conclusions:**

Inflatable occlusion can increase middle-aged subjects’ blood perfusion coherence value of the forearm. However, there is no specificity in meridian location.

## Background

In Traditional Chinese Medical (TCM) theory, the acupuncture effect is based on the integrity function of the meridian, which in turn is the core concept of metaphysical acupuncture theory [1]. However, it is difficult to evaluate the activation of meridians. Until now, the broad consensus in meridian studies has been the lower impedance along the meridians [2,3]. Usually, the impedance of the skin is proportional to the interstitial fluid volume arising from microcirculation; thus, microcirculation may be an index for meridian activation. In addition, some studies have suggested that the meridian system may contain a continuous channel [4] to facilitate signal transport in peripheral tissues [5,6], which provides further evidence for the relationship between microcirculation and meridians [7]. Although much attention has been focused on improving the understanding of the relationship between meridians and microcirculation [8-10], the synchronous characteristics of blood perfusion along meridians are still unknown. Thus, the purpose of the current study was to quantify the frequency-dependent covariation of blood perfusion along meridians using the cross-spectral analyses (coherence) method.

Previous studies had indicated that blood-flow oscillations at frequencies from 0.009 to 1.6 Hz might reflect various physiological rhythms [11], which can be separated into five different frequency bands in frequency domain [12-15]. Thus the blood perfusion signal contains a mixture of the influence of endothelial activity, neurogenic activity, intrinsic myogenic activity, respiration, and heart rate. Different frequency bands show different clinical implications. On the other hand, coherence is a measure of the correlation of two signals. If the investigated two signals are assumed to be stationary, then the coherence can be calculated to estimate the correlation of the signals at different frequencies. Coherence analysis has been used widely in biosignal analysis, not only in EEG and EMG [16], but also in the blood flow analysis [17]. So coherence is a suitable method for blood perfusion analysis.

## Methods

### Ethics statement

This study was reviewed and approved by the Institutional Review Board at the Institute of Acupuncture & Moxibustion, China Academy of Chinese Medical Sciences (CACMS). Each subject provided informed consent and had an adequate understanding of the procedure and purpose of this study.

### Subjects

Fifteen healthy young subjects(Group I,n = 15) aged from 21 to 30 (25.53 ± 2.20) and fifteen healthy middle age subjects(Group II,n = 15) aged from 46 to 57(50.07 ± 3.37) were recruited for this study. All younger subjects were students from the CACMS and Beijing University of Chinese Medicine. All middle-aged subjects were from the CACMS. All subjects had no past history of disease, and had not taken any medication in the past 6 months (Table 1).

**Table 1:** Main characteristics of the studied individuals

**Characteristics**	**Group I**	**Group II**	**t-value**	**P-value**
Gender (Femal/man)	10/5	12/3		
Age (Years)	25.53 ± 2.20(21–30)	50.07 ± 3.37(46–57)	−23.614	0
Systolic pressure (mmHg)	103.27 ± 17.77(70–140)	114.67 ± 13.02(90–140)	−2.004	0.055
Diastolic pressure (mmHg)	71.13 ± 10.04(60–90)	80.00 ± 11.18(60–90)	−2.286	0.030
Heart rate (BPM)	69.134 ± 9.924(52–86)	66.33 ± 5.99(56–76)	0.936	0.358
High (cm)	163.07 ± 4.067(157–172)	162.87 ± 5.11(153–173)	0.119	0.906
Body weight (kg)	56.47 ± 5.597(47–70)	61.60 ± 8.07(50–77)	−2.025	0.520
Body mass index (BMI)	21.21 ± 1.68(18.36-24.34)	23.16 ± 2.24(20.20-27.34)	−2.694	0.012

### Protocol for measurement of mean blood flux

Upon arrival at the laboratory, subjects were placed in a temperature-controlled room (24-26°C) for 60 min. Measurements of skin blood perfusion were carried out using full-field laser blood perfusion imaging (FLPI, Moor Instruments, Devon, UK). Before recording, the left arm was immobilized to ensure positioning and during the recording, the subject remains supine position. The single point (32–36 pixels) measurements model was selected.

**Figure 1:**
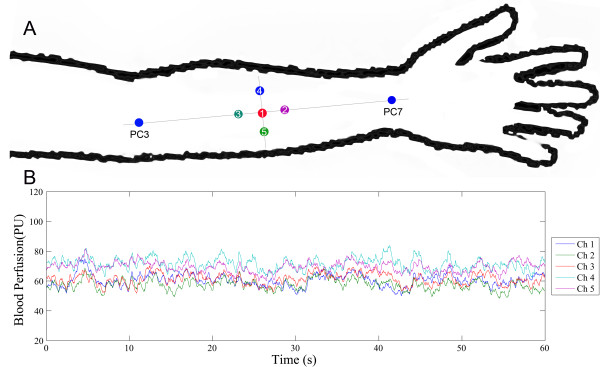
**Illustration of the study design. (A)** the measure points selection; **(B)** the original blood perfusion signal. PC, Pericardium Meridian; PC3, Quze acupoint; PC7, Daling acupoint. 1, 2, 3, 4, 5 represent the measurement points of Ch 1, Ch 2, Ch 3, Ch 4, Ch 5, respectively.

In the TCM theory, both acupoint and meridian are core concept to explain the acupuncture effect. According to the meridian theory, the acupuncture information might be transported along the meridian continuously. So the meridian channel has its’ own specificity compared with the parameridian area. Our aim is to investigate the meridian specificity with blood perfusion coherence analysis, but not the acupoint specificity. In this study, a total of 5 points (described as channels in the Moor FLPI recording software) were used for recordings (Figure 1A). Point 1 (Channel 1, Ch1) was the midpoint of Quze (PC3) and Daling (PC7), all of which belong to the pericardium meridian. Point 4 (Channel 4, Ch4) was located on the radial side of the pericardium meridian, while Point 5 (Channel 5, Ch5) was located on the ulnar side. Point 2 (Channel 2, Ch2) and Point 3 (Channel 3, Ch3) were both located on the pericardium meridian, Ch3 on the proximal side and Ch2 on the distal side. Ch1 and the other four channels were equidistant.

For every acupunctist, the PC meridian in forearm is defined by the PC7 and PC3, thus the recording point 1, 2 and 3 belong to the PC meridian. Recording point 4 and 5 obviously don’t belong to the PC meridian. On the other hand, how far the distance between the recording points will be another considering factor. In this study, the diameter was quartered by the point 4, point 1 and point 5. Thus the distance from point 1 to other four points is equal, and the bias due to the different distance between recording points will be ruled out.

25-Hz sample rate, an 8.33-ms exposure time, and 0.5 s time constant were set. After skin blood perfusion had been recorded for 30 min, skin ischemia was induced by inflating a pneumatic cuff just above the elbow, with pressure maintained at 30 mmHg for 3 min. After this time, the pneumatic cuff was instantaneously deflated, and skin blood perfusion was recorded for another 30 min. During the experiment, the laboratory room was kept in dark lighting conditions, and the protocol for measurement operation was strictly adhered to. The measurement process is illustrated in Figure 1A, and the original signal shown in Figure 1B.

### Data analysis

To calculate the coherence between Ch1 and the other four channels, the coherence value was estimated as follows [18,19]:

$$C_{\mathrm{xy}}\left(f\right)=\frac{{\left|P_{\mathrm{xy}}\left(f\right)\right|}^{2}}{P_{\mathrm{xx}}\left(f\right)P_{\mathrm{yy}}\left(f\right)}$$

This equation obtains the magnitude squared coherence estimate *C*_*xy*_(f) of the input signals *x* and *y* using Welch’s averaged, modified periodogram method. The value of *C*_*xy*_(f) is between 0 and 1 and indicates how well *x* corresponds to *y* at each frequency. Computations were performed using the toolbox as follows in the MATLAB software (R2011a).

$$\left[C_{\mathrm{xy}},F\right]=\mathrm{mscohere}\left(x,y,\mathrm{window},\mathrm{noverlap},\mathrm{nfft},\mathrm{fs}\right)$$

In this study, *x* represents the Ch1 measurement value of blood perfusion, and *y*_i_(i = 2,3,4,5) represents the signal of Ch2, Ch3, Ch4, and Ch5, respectively. window = hanning(1024), noverlap = 512, nfft = 20000, and fs = 25. *C*_*xy*_ is the coherence value at *F* frequency. Then *F* was segmented and 5 frequency bands (0.0095-0.02Hz, 0.02-0.06Hz, 0.06-0.15Hz, 0.15-0.4Hz, 0.4-1.6Hz) were extracted according to the reference [20]. The mean *C*_*xy*_ for every frequency band was calculated.

### Statistical analysis

Group differences were analyzed using SPSS software (Version 13.0, SPSS Inc., Chicago, IL). The coherence values of blood perfusion in every frequency interval were expressed as mean ± SD. Statistical analysis was performed using paired *t*-tests. Values of p < 0.05 were considered statistically significant.

## Results

**Figure 2:**
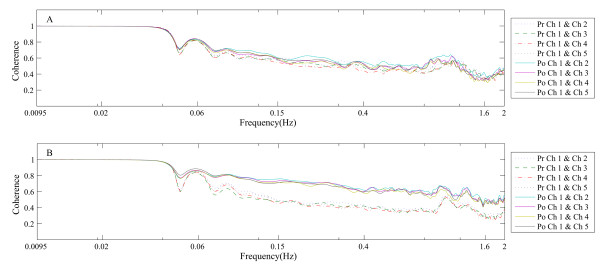
**Coherence between channel 1 and other four channels. (A)** Young subject group (n = 15); **(B)** the middle-aged group (n = 15); Pre, pre-inflatable occlusion; Po, post-inflatable occlusion; Ch, Channel.

In this study, a total of 30 subjects were included in the final analyses. Figure 2 shows that irrespective of the coherence value associated with the measurement point, or the age group, the lower the frequency the higher the coherence value obtained. Additionally, we found that with an increase in frequency, the coherence value decreased.

In young subjects (Group I), the coherence value between Channel 1 and the other four channels was unchanged in each frequency interval before and after skin ischemia (Figure 2A, Table 2). However, in middle-aged subjects (Group II), the coherence value between Channel 1 and the other four channels increased significantly after skin ischemia in frequency intervals 3, 4, and 5 (Figure 2B, Table 3).

**Table 2:** Coherence values of different frequency intervals before and after inflatable occlusion in Group I (n = 15)

**Frequency intervals(Hz)**	**Channels**	**Pre**	**Post**
**FR1 (0.0095-0.02)**	Channel 1 to 2	0.9987 ± 0.0009	0.9983 ± 0.0014
	Channel 1 to 3	0.9987 ± 0.0010	0.9986 ± 0.0010
	Channel 1 to 4	0.9982 ± 0.0022	0.9982 ± 0.0014
	Channel 1 to 5	0.9985 ± 0.0016	0.9984 ± 0.0016
**FR2 (0.02-0.06)**	Channel 1 to 2	0.9059 ± 0.0394	0.9093 ± 0.0656
	Channel 1 to 3	0.8992 ± 0.0439	0.9117 ± 0.0484
	Channel 1 to 4	0.9003 ± 0.0431	0.9051 ± 0.0482
	Channel 1 to 5	0.9003 ± 0.0400	0.9074 ± 0.0508
**FR3 (0.06-0.15)**	Channel 1 to 2	0.6238 ± 0.1984	0.6876 ± 0.2108
	Channel 1 to 3	0.6198 ± 0.1849	0.6611 ± 0.1994
	Channel 1 to 4	0.6165 ± 0.1959	0.6671 ± 0.1924
	Channel 1 to 5	0.6364 ± 0.1789	0.6724 ± 0.1959
**FR4 (0.15-0.4)**	Channel 1 to 2	0.5478 ± 0.2663	0.5747 ± 0.2851
	Channel 1 to 3	0.5229 ± 0.2664	0.5375 ± 0.2845
	Channel 1 to 4	0.4903 ± 0.2695	0.5292 ± 0.2956
	Channel 1 to 5	0.5158 ± 0.2674	0.5527 ± 0.2562
**FR5 (0.4-1.6)**	Channel 1 to 2	0.4874 ± 0.2334	0.5111 ± 0.2604
	Channel 1 to 3	0.4680 ± 0.2409	0.4749 ± 0.2545
	Channel 1 to 4	0.4475 ± 0.2358	0.4519 ± 0.2433
	Channel 1 to 5	0.4586 ± 0.2294	0.4661 ± 0.2398

Pre, pre-inflatable occlusion. Post, post-inflatable occlusion. FR, frequency interval. There was no significant difference between Pro- and Post-occlusion.

**Table 3:** Coherence values of different frequency intervals before and after inflatable occlusion in Group II (n = 15)

**Frequency intervals(Hz)**	**Channels**	**Pre**	**Post**
**FR1 (0.0095-0.02)**	Channel 1 to 2	0.9988 ± 0.0006	0.9983 ± 0.0019
	Channel 1 to 3	0.9987 ± 0.0009	0.9984 ± 0.0015
	Channel 1 to 4	0.9987 ± 0.0006	0.9979 ± 0.0031
	Channel 1 to 5	0.9987 ± 0.0009	0.9984 ± 0.0011
**FR2 (0.02-0.06)**	Channel 1 to 2	0.9069 ± 0.0434	0.9221 ± 0.0577
	Channel 1 to 3	0.9044 ± 0.0431	0.9287 ± 0.0452
	Channel 1 to 4	0.9011 ± 0.0587	0.9247 ± 0.0486
	Channel 1 to 5	0.9047 ± 0.0417	0.9365 ± 0.0365*
**FR3 (0.06-0.15)**	Channel 1 to 2	0.6034 ± 0.2444	0.7913 ± 0.1889*
	Channel 1 to 3	0.5528 ± 0.2543	0.7806 ± 0.1855*
	Channel 1 to 4	0.5673 ± 0.2608	0.7650 ± 0.1974*
	Channel 1 to 5	0.5940 ± 0.2276	0.7671 ± 0.1852*
**FR4 (0.15-0.4)**	Channel 1 to 2	0.4393 ± 0.2808	0.7206 ± 0.2266**
	Channel 1 to 3	0.4225 ± 0.2846	0.7103 ± 0.2323**
	Channel 1 to 4	0.4103 ± 0.2826	0.6950 ± 0.2302**
	Channel 1 to 5	0.4035 ± 0.2904	0.6930 ± 0.2335**
**FR5 (0.4-1.6)**	Channel 1 to 2	0.3939 ± 0.2864	0.6265 ± 0.2242*
	Channel 1 to 3	0.3722 ± 0.2754	0.6070 ± 0.2320*
	Channel 1 to 4	0.3739 ± 0.2800	0.5782 ± 0.2147*
	Channel 1 to 5	0.3638 ± 0.2829	0.5889 ± 0.2281*

Pre, pre-inflatable occlusion. Post, post-inflatable occlusion. FR, frequency interval.

*, p < 0.05, **, p < 0.01, Pre vs. Post.

## Discussion

Recently, increasing attention has been focused on the relationship between acupuncture and circulation [21-23]. Previous studies have indicated that acupuncture can not only affect the general circulation [24,25], but also affect skin microcirculation [20,22,26,27]. In addition, under resting conditions the mean blood perfusion was higher at the acupoints compared with surrounding tissues [10]. Moreover, adequate acupoint stimulation resulted in increased blood perfusion at this point, whereas blood perfusion of the non-acupoint only changed slightly after the same acupuncture stimulation [28,29]. Importantly, studies have suggested that the skin microvasculature mirrors the vascular function of other parts of the body [30-32], which is in line with the basic standpoints of acupuncture theory.

Many studies have indicated that ageing is associated with attenuated vasodilator responses of skin microcirculation as a result of a variety of stimuli [33-35]. In this study, we observed that occlusion resulted in different coherence values in middle-aged subjects. However, the coherence value changes were in frequency intervals 3, 4, and 5, which is not consistent with age-associated endothelial dysfunction [36]. In this regard, we believe that there are two possibilities. One is that the same stimuli for different age groups resulted in the different results. For young person, the stimuli of 30 mmHg inflating occlusion last 3 min couldn’t disturbance the normal function, while for middle-aged subjects, the same stimuli might be a up threshold stimuli and the body must be adaptive to the stress. The other is that both endothelial and neurogenic activity might only response to the specific stimuli, and the changes of intrinsic myogenic activity, respiration, and heart rate resulted from nonspecific stress.

In this study, Channels 1, 2, and 3 were located in the pericardial meridian, while Channels 4 and 5 were located in the parameridian position. According to acupuncture theory, the blood perfusion of Channels 1, 2, and 3 are all closely related, which could be the reason for the higher coherence values of these channels, whereas the coherence values between Channels 1 and 4, and Channels 1 and 5 should be lower. However, our results do not support this conjecture. We believe that one possible reason for this is that differences between the meridian and the parameridian positions do not exist. The other possible reason is that the stimulus in this study is lack of specificity. In the future, we should apply the specific stimuli at the different acupoint and then explore the coherence difference between M-M and M-P.

## Conclusions

Skin ischemia can increase blood perfusion coherence values in the forearms of middle-aged subjects; however, there is no specificity in meridian location.

## Competing interests

The authors declare that they have no competing interests.

## Author’s contributions

WG carried out the design and participated in data collection. TY, JS, and ZW participated in data collection. ZW led the design and participated in data collection. All authors read and approved the final manuscript.

## Pre-publication history

The pre-publication history for this paper can be accessed here:

http://www.biomedcentral.com/1472-6882/13/327/prepub
